# Robotic Lateral Pelvic Lymph Node Dissection in Rectal Cancer: A Feasibility Study from a European Centre

**DOI:** 10.3390/jcm13010090

**Published:** 2023-12-23

**Authors:** Tou Pin Chang, Oroog Ali, Kostas Tsimogiannis, Giuseppe S. Sica, Jim S. Khan

**Affiliations:** 1 Epsom and St. Helier University Hospitals NHS Trust, Carshalton SM5 1AA, UK; toupin.chang@nhs.net; 2 Gateshead Health NHS Foundation Trust, Gateshead NE9 6SX, UK; oroog.ali@nhs.net; 3 Department of Colorectal Surgery, Victory Institute of Minimal Access Surgery, Queen Alexandra Hospital, Portsmouth Hospitals University NHS Trust, Portsmouth PO6 3LY, UK; ktsimogiannis@gmail.com; 4 Department of Surgery, Minimally Invasive Unit, Università degli Studi di Roma, Tor Vergata Hospital, Viale Oxford 81, 00133 Rome, Italy; sigisica@gmail.com

**Keywords:** robotic rectal cancer, lateral pelvic lymph node dissection, minimally invasive surgery, robotic colorectal

## Abstract

Introduction: The role of robotic lateral pelvic lymph node dissection (LPLND) for lateral pelvic nodal disease (LPND) in rectal cancer has yet to be investigated in the Western hemisphere. This study aims to investigate the safety and feasibility of robotic LPLND by utilising a well-established totally robotic TME protocol. Methods: We conducted a retrospective study on 17 consecutive patients who underwent robotic LPLND for LPND ± TME for rectal cancer between 2015 and 2021. A single docking totally robotic approach from the left hip with full splenic mobilisation was performed using the X/Xi da Vinci platform. All patients underwent a tri-compartmental robotic en bloc excision of LPND with preservation of the obturator nerve and pelvic nerve plexus, leaving a well-skeletonised internal iliac vessel and its branches. Results: The median operative time was 280 min, which was 40 min longer than our standard robotic TME. The median BMI was 26, and there were no conversions. The median inpatient stay was 7 days with no Clavien-Dindo > 3 complications. One patient (6%) developed local recurrence and metastatic disease within 5 months. The proportion of histologically confirmed LPND was 41%, of which 94% were well to moderately differentiated adenocarcinoma. Median pre-operative lateral pelvic node size was significantly higher in positive nodes (14 mm vs. 8 mm (*p* = 0.01)). All patients had clear resection margins on histology. Discussion: Robotic LPLND is safe and feasible with good peri-operative and short-term outcomes, with the ergonomic advantages of a robotic TME docking protocol readily transferrable in LPLND.

## 1. Introduction

Total mesorectal excision (TME) is the gold standard surgical treatment for mid to low rectal cancer [1]. Approximately 10–25% of patients with rectal cancer will have lateral pelvic nodal disease (LPND), whereby TME alone is insufficient to achieve disease control [2,3]. Lateral pelvic lymph node dissection (LPLND) with TME has been the routine management strategy for LPND in the Far East [4], but this practice is not widely adopted in the West as LPND is often viewed as a metastatic entity rather than locally advanced disease and thereby receive neoadjuvant treatment instead [5,6,7]. However, recent evidence has demonstrated that patients with LPND who underwent neoadjuvant treatment followed by TME without LPLND have a higher local recurrence rate of up to 30% compared to those who underwent LPLND [8]. Consequently, there is now increasing interest across the globe to acquire the technical skills and minimally invasive strategies in LPLND to address this major cause of local recurrence [9].

Robotic surgery offers a stable operative platform integrated with three-dimensional (3D) visualisation, enhanced dexterity and articulation of instruments that enable deep pelvic tissue dissection pertinent to LPLND to be carried out with high accuracy and precision. Yamaguchi and colleagues have demonstrated that robotic LPLND was associated with lower blood loss and post-operative complications compared to open surgery with comparable long-term overall outcomes [10]. There is limited evidence comparing the robotic approach with laparoscopic LPLND, but preliminary results have suggested lower blood loss and post-operative complications in the robotic approach with comparable local recurrence rates [11]. The role of robotic surgery in LPLND in the Western hemisphere has yet to be investigated, and we postulate that the established advantages and attained technical skillset of robotic TME in the Western population with higher body mass index (BMI) and narrow pelvis, especially in men, is readily transferrable and pertinent in LPLND.

The aim of this study is to investigate the safety and feasibility of robotic LPLND for LPND in a high-volume rectal cancer unit by utilising a well-established TME protocol, careful case selection and a systematic tri-compartmental dissection approach in LPLND [9,12].

## 2. Methods

### 2.1. Patient Selection

**Figure 1:**
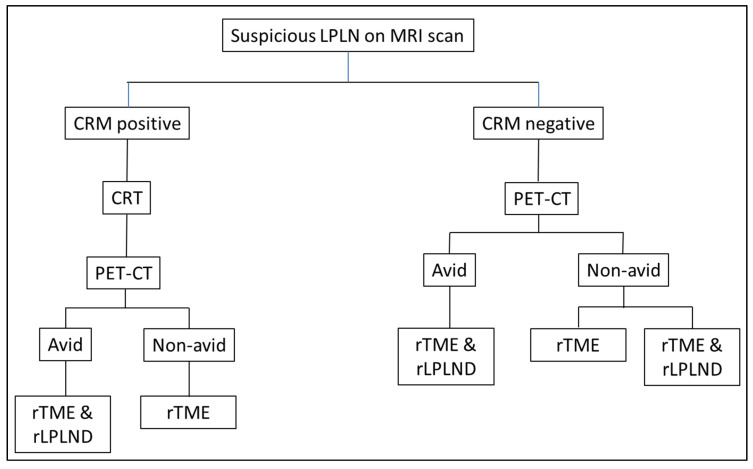
Flow chart of patient selection for robotic LPLND; CRM: Circumferential resection margin; CRT: chemoradiotherapy/chemotherapy; PET-CT: position emission tomography; rTME: robotic total mesorectal excision; rLPLND: robotic lateral pelvic lymph node dissection.

This is a retrospective analysis of a prospectively maintained rectal cancer database at our institution. LPLND were performed on patients with pre-operative magnetic resonance imaging (MRI), confirmed enlarged and suspicious-looking LPLNs and in those who had persistent LPLN avidity demonstrated on Position Emission Tomography (PET-CT) following neoadjuvant chemoradiotherapy treatment because of locally advanced rectal cancer or suspicious lateral lymph nodes. (Figure 1). Patients who underwent LPLND for suspicious-looking LPLNs following previous rectal cancer surgery were also included in this study. All patients had their surgeries performed by a single senior colorectal surgeon (J.S.K.) with significant experience in minimal access and robotic surgery. We retrieved the relevant patient demographics, pathological characteristics, operative findings and post-operative outcomes for data analysis. All statistical analysis was performed using the statistical software package SPSS 21.0 (SPSS, Chicago, IL, USA).

### 2.2. Robotic Setup

**Figure 2:**
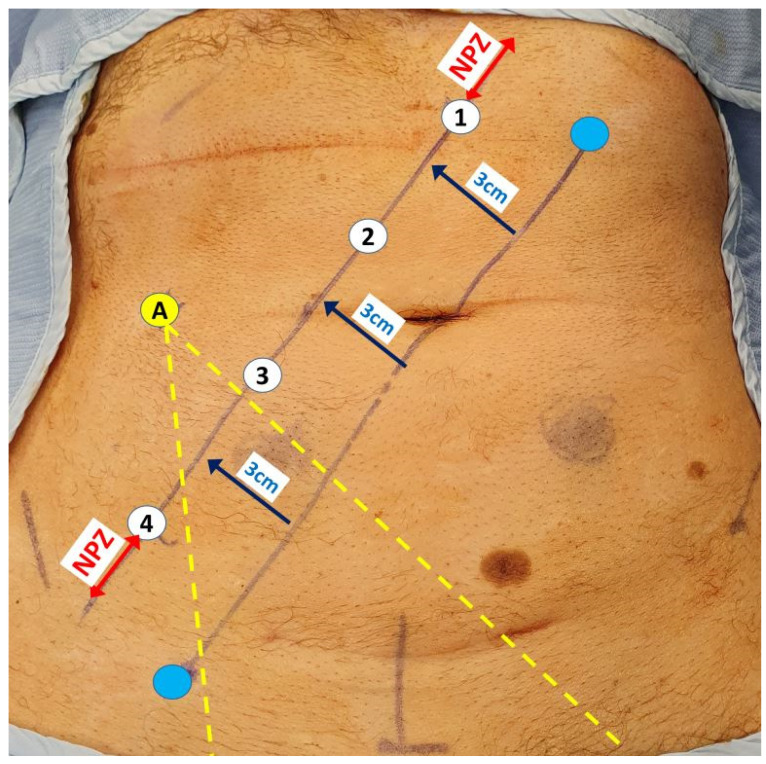
Robotic port placements (numbered circles) for LPLND ± TME along a 3 cm lateralised diagonal line from an initial imaginary line between the mid-inguinal point (blue circle) and the left costal margin (blue circle) lateralised at 8 cm from the midline. The assistant port (A) is placed at least a fist breadth away from the robotic ports to allow optimal instrument entry to the pelvis between the third and fourth robotic ports (yellow dotted lines). The ‘No port zone’ (NPZ) denotes the area within two fingers breadth from adjacent bony landmarks, which should be devoid of any ports.

All procedures were carried out using a single docking totally robotic approach from the left hip with full splenic mobilisation as previously described using either an X or Xi da Vinci robotic platform [13] (Intuitive Surgical Inc., Sunnyvale, CA, USA) with no additional ports required (Figure 2). In cases of TME with LPLND, the LPLND component was performed following completion of TME and rectal transection with 1–2 firings of Sureform^®^ 60 mm linear robotic stapler (Intuitive Surgical Inc., Sunnyvale, CA, USA) [14], starting with the left LPLND if bilateral dissections were clinically indicated, followed by primary colorectal or coloanal anastomosis and defunctioning ileostomy where appropriate. All cases were performed with an experienced advanced nurse practitioner or a senior clinical fellow as bedside assistants who have completed their mandatory da Vinci^®^ virtual and hands-on training modules. Patients were given mechanical bowel preparation with Picolax and received antibiotics and VTE prophylaxis. Enhanced recovery pathway was the standard of care post-operatively.

### 2.3. Anatomical Details and Surgical Technique

**Figure 3:**
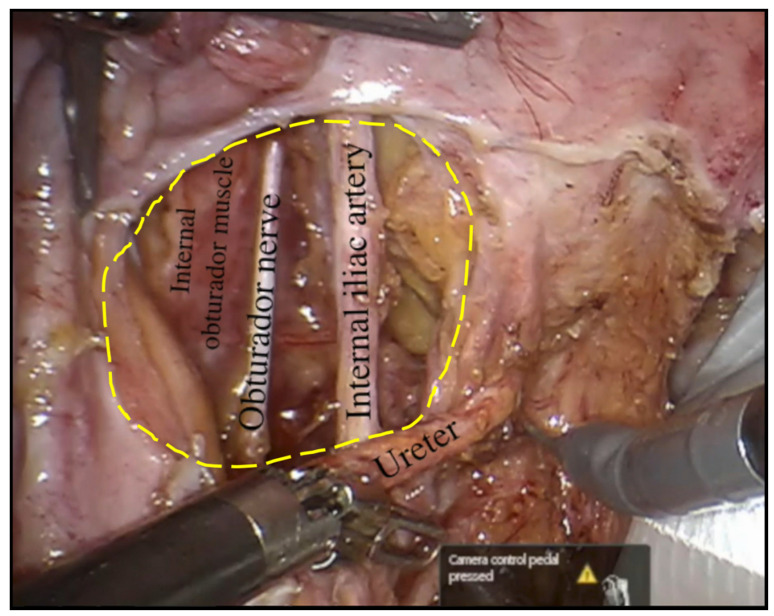
The obturator fossa in the left pelvic side wall following completion of robotic LPLND with the internal obturator muscle depicting the lateral boundary of the fossa at its depth and the central compartment, which consists of the left obturator nerve and left internal iliac artery with the left ureter returned to its anatomical position.

**Figure 4:**
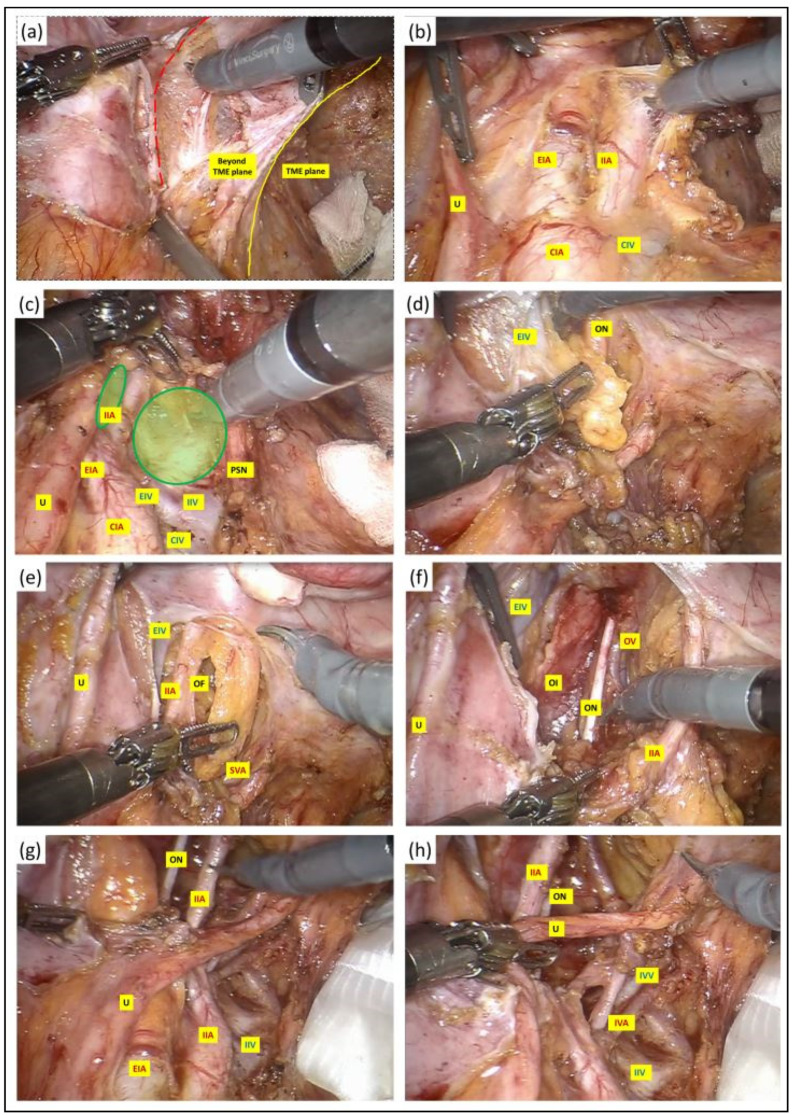
(**a**) A vertical peritoneal incision was made at the medial umbilical fold, and this was extended to the level of common iliac artery bifurcation. The dissection was continued along the external iliac vessels (red dotted line) and the lymph nodes along these vessels were excised in their entirety with their surrounding lymphatic tissue package kept intact. (**b**–**d**) Once the external iliac vein had been dissected free, the third robotic arm with a double fenestrated forceps was deployed against the ureter for gentle retraction to facilitate entry into the obturator fossa between the internal and external iliac vessels (green circles), clearing the lymphatic package in a craniocaudal fashion. The extent of the dissection anteriorly was at the level where the plane started to fuse with the prevesical tissue and the femoral canal. Further division of nodal tissues at this stage was performed between clips to prevent lymphatic leak. (**e**) As the dissection approached the depth of the obturator fossa, the obturator vessels and nerve were encountered medial to the external iliac vein and lateral to the superior vesical artery. We paid careful attention not to injure or divide the obturator nerve to prevent post-operative neuropathy. The obturator artery and vein, on the other hand, were divided between clips if clinically indicated. (**f**) The nodal dissection was continued along the internal iliac artery and its anterior divisions to expose the superior and inferior vesical, obturator and umbilical artery from the anterior branch and along the posterior branch to allow en bloc excision of the pathological lymph node mass with its surrounding lymphatic tissue package kept intact, and this was retrieved in a bag. The lateral boundary of dissection at the depth of the obturator fossa was the obturator internus, whereas medially, the dissection at this depth continued into the TME plane that was created at the earlier stage. (**g**,**h**) Following completion of LPLND, the remaining structures in the pelvic side wall consist of well-skeletonised internal iliac vessels and their branches, the obturator nerve and the pelvic nerve plexus preserved in its entirety with no intervening residual fat or nodal tissues. U: ureter; CIA: common iliac artery; EIA: external iliac artery; IIA: internal iliac artery; CIV: common iliac vein; EIV: external iliac vein; PSN: presacral nerve plexus; OF: obturator fossa; IVA: inferior vesicle artery; IVV: inferior vesicle vein; OI: obturator internus; ON: obturator nerve.

The commonest site of LPLN metastasis in rectal cancer is in the obturator fossa or alongside the internal iliac artery branches. LPLND involves en bloc excision of all the nodal tissues incorporating the pathological nodes in a systematic fashion alongside three facial compartments: the lateral compartment, which is bounded laterally by the psoas and internal obturator muscle; the medial compartment, which is bounded medially by the ureter and the pelvic plexus; and the central compartment, which is composed of the internal iliac vessels and the sciatic nerve (Figure 3). The key operative steps pertaining to the dissection, identification and preservation of critical neurovascular structures of the pelvic side wall are described in Figure 4.

## 3. Results

Seventeen consecutive patients underwent robotic LPLND with or without TME for rectal cancer over a six-year period between 2015 and 2021. The median BMI was 26, with a median ASA grade of 2 (Table 1). Approximately one-third of patients received neoadjuvant treatment in the form of either chemotherapy or concurrent chemoradiotherapy. The median tumour height was 6 cm. Eighty-eight percent of LPLND were performed unilaterally, and 59% were carried out in conjunction with TME, primary anastomosis and defunctioning ileostomy. The majority of the remaining 35% of those who underwent LPLND only had previously undergone TME surgery for rectal cancer, albeit one patient had a diagnostic LPLND for a benign lymphoproliferative disorder. The intraoperative blood loss was minimal, and no patients received peri-operative blood transfusion. Two-thirds of patients had a trans-abdominal drain inserted for 48 to 72 h, and there were no post-operative collections following its removal. There were no conversions, and the median operative time was 280 min, which was approximately 40 min longer than our standard robotic TME. The majority of our patients spent a night at the surgical high dependency unit (sHDU), and the median inpatient stay was seven days. There was no unplanned 30-day return to theatre or Clavien-Dindo 3 and above complications. Two patients were readmitted within 30 days post-discharge with ileus and high stoma output, respectively, which were managed conservatively. The median follow-up was 18 months (1–73). One patient (6%) developed local recurrence and distant metastatic disease and died within five months.

The proportion of patients with positive lymph node(s) (LNs) in their LPLND specimens was 41% (Table 2). These were predominantly adenocarcinoma (94%), of which all were of well to moderately differentiated grades. The median number of LNs harvested was 8, with a median LPLND specimen volume of 48,000 mm^3^. All patients who had robotic TME or APER surgery in conjunction with robotic LPLND had clear resection margins on their primary tumour on histology. The pathological characteristics for LN positivity status from LPLND specimens are outlined in Table 2. The pre-operative LPND size was significantly larger in those with LN-positive disease (*p* = 0.01), and there were no significant differences in the remaining tumour characteristics variables (Table 3). All patients had a successful removal of the urinary catheter, and although no detailed functional assessments were carried out, there were no reports of significant urinary or sexual dysfunction at follow-up.

## 4. Discussion

This study reports on the use of a robotic platform to perform LPLND for lateral nodal disease in rectal cancer. By utilizing the da Vinci^®^ Surgical System as a robotic platform, we have demonstrated that robotic LPLND is a safe and feasible procedure with the tri-compartmental dissection of the lateral pelvic side wall readily reproducible, consistent with published technical descriptions from the East [9,12].

The overall peri-operative clinical outcomes and length of stay were comparable with our published series on standard robotic TME, with the addition of 40 min to the overall operative time being the overt discriminating difference [13]. From the pathological context, the lymph node positivity yield of 41% on LPLND specimens is similar to those reported by Konishi et al., owing to the diagnostic limitations of pre-operative MRI and PET-CT as staging modalities [9]. It is well recognised that low rectal tumours with poor differentiation or extramural venous invasion (EMVI) are more likely to have LPND. Whilst this study was not designed to evaluate the short- and long-term outcomes following LPLND, our preliminary data suggested that the incidence of local recurrence had been low, albeit one patient succumbed to widespread metastatic disease progression. Additionally, there was no reported incidence of new contralateral lateral nodal disease in those that underwent unilateral LPLND and, therefore, might suggest that targeted unilateral LPLND might be considered reasonable, thus minimising the theoretical risk of autonomic nerve dysfunction unless bilateral nodal disease were conspicuous at pre-operative staging where bilateral LPLND is warranted.

It is worth noting that LPLND is often performed in the setting of irradiated tissues or previous radical pelvic surgery such as TME, whereby a significant loss of tissue domains or planes is often encountered. One of the major advantages of a robotic platform in these circumstances is its ability to deliver an enhanced three-dimensional (3D) visualisation and magnification of critical neurovascular side wall structures in the deep pelvis through a stable operative workflow that augments our depth of perception and tissue differentiation [15,16]. Equipped with the mechatronically enhanced robotic Endo Wrists^®^, it provides enhanced articulation beyond the limits of human wrist movements and laparoscopic instruments. It effectively eliminates human hand tremor, drift and fatigue, thus facilitating superior operative dexterity with augmented precision during tissue plane dissection whilst preserving stable and reliable tissue retraction. The ergonomic utility of the port placement in accordance with the Portsmouth protocol [13] for robotic TME was pertinent for both lateralities of LPLND, with no requirements for additional port placements, re-docking of the robot or reported incidence of instrument clashes. Our cumulative experience of the perceived ergonomic benefits of robotic LPLND was consistent with those reported from high-volume centres in the East [12] and beyond TME surgery, such as multivisceral exenteration whereby enhanced visualisation and dexterity are necessary requisites for high-quality oncological outcomes [16].

In spite, however, of the various technical advantages of robotic surgery, it is imperative that a comprehensive understanding of the lateral pelvic side wall anatomy and prior laparoscopic experience in TME and LPLND are to be considered as essential pre-requisites prior to embarking onto robotic LPLND cases which often necessitate experienced proctoring or remote telementoring during the early phases of the learning curve. The projected number of cases in this phase remains unknown at this stage, but we do envisage that with the recent wider acquisition of robotic TME skills and experience, a rapid establishment of a larger pool of proficient robotic surgeons will be equipped with the armamentarium to develop further interests in robotic LPLND and thus yield meaningful data on the learning curve characteristics. Given the relatively low case volumes at this stage, it is plausible to hypothesise that the establishment of a robotic LPLND service and training programme will require a centralisation model with established regional and national pathways of referral to accelerate this learning curve. Drawing from our experience, approximately half of our case volumes of robotic LPLND in recent years were indeed collated from external referrals with our local, regional cancer network.

At present, the plethora of experience in robotic LPLND for rectal cancer is derived primarily from case series and non-randomised comparative studies from the East with safety and feasibility profiles comparable to laparoscopic LPLND. Whilst the case volume in the West is notably lower, strategies to circumvent this limitation are likely to require the creation of a national LPLND database that would enable a fast track and collaborative approach in the collation of tumour biology characteristics, quality control, oncological and functional outcomes across all operative approaches. This will enable the concurrent collection of appraisable robotic data outcomes in line with an established national dataset collection tool. Additionally, the creation of subgroup committees with LPLND interest through national surgical associations could potentially facilitate constructive open-table dialogues on the identification of potential barriers in the uptake of robotic LPLND and the implementation of resolution strategies.

We wish to highlight that our operative times with robotic TME have traditionally been significantly shorter when compared to laparoscopic TME, and this is largely attributable to our consistently high volume of robotic TME, which were invaluable ingredients to the development of our highly experienced robotic operating theatre team and efficient peri-operative workflow pathways [13]. This is indeed contrary to some studies, where the operative times for robotic TME were longer, but it is in part attributable to a significant proportion of robotic surgeons still in the early phases of their learning curve [15]. It is, therefore, conceivable that promising benefits of robotic LPLND are likely to be reproducible in high-volume robotic TME centres whereby the attained robotic proficiency skills in the pelvis are readily transferrable to LPLND.

Whilst we acknowledge the controversies pertaining to the role of LPLND in the West, it is beyond the scope of this study to debate the relative merits of LPLND in its present state with a very small number of surgeons with the appropriate experience to deliver this service. Additionally, the paucity of clinical experience with robotic LPLND in the West meant that our study had to include a relatively small and heterogeneous group of patients to demonstrate its safety and feasibility in the hope that it would serve as an initial evidence-based platform to support future well-powered and robust studies of robotic LPLND. However, we postulate that some of the true benefits of LPLND have yet to be explored in the West, and if the harvested benefits of robotic assistance could be translated into superior oncological and preservation of functional outcomes, considerable investment in a robotic approach should be considered, therefore substantially justifying the marginally increased operating time and its associated additional costs [15]. To that end, further prospective multi-centred collaborative-based studies on safety, efficacies, operative, short-term oncological and functional outcomes comparing robotic with laparoscopic and open approaches in LPLND constitute the next immediate stage of research priorities.

## Figures and Tables

**Table 1:** Baseline characteristics and peri-operative outcomes. Values are presented as numbers (%) or median (range). ASA: American Society of Anesthesiologists; AV: anal verge; CRA: colorectal anastomosis; CAA: coloanal anastomosis; DI: defunctioning ileostomy; APER: abdominoperineal excision of the rectum. HDU: high dependency unit; TPN: total parenteral nutrition.

Age (year)	63 (27–79)
Sex - Male - Female	12 (71) 5 (29)
Body mass index (kg/m^2^)	26 (20–40)
Neoadjuvant treatment	6 (35)
- Chemotherapy - Concurrent chemoradiotherapy - Chemotherapy and short-course radiotherapy	3 (18) 2 (12) 1 (6)
ASA grade	2 (2–3)
Tumour distance from the anal verge (cm)	6 (4–10)
Laterality - Unilateral - Bilateral	15 (88) 2 (12)
Operative procedure - TME, CRA/CAA, DI and LPLND - APER and LPLND - LPLND	10 (59) 1 (6) 6 (35)
Length of extraction site (cm)	5 (4–6)
Intraoperative blood loss (ml)	20 (0–100)
Operative time (minutes)	280 (80–380)
Pelvic drain - Yes - No	11 (65) 6 (35)
Peri-operative blood transfusion	0 (0)
Length of HDU stay (days)	1 (1–9)
Length of inpatient stay (days)	7 (3–20)
Length of HDU stay (days)	2 (1–9)
Post-operative complication	3 (17)
- Lymphatic leak - Prolonged ileus requiring TPN - Urinary retention	1 (6) 1 (6) 1 (6)
Conversion - Robotic to laparoscopic - Robotic to open	0 (0) 0 (0)
30-day return to theatre	0 (0)
Unplanned 30-day re-admission	2 (12)
- Prolonged ileus - High stoma output	1 (6) 1 (6)
Time to ileostomy reversal (months)	7 (6–24)
Adjuvant chemotherapy	9 (17)
Follow-up (months)	18 (1–73)
Six-month local recurrence	1 (6)
- Time to recurrence (months)	4

**Table 2:** Pathological outcomes. Values are presented as numbers (%) or median (range). R0: microscopically margin-negative resection; R1: microscopic residual tumour; R2: macroscopic residual tumour.

Number of LPLND-positive cases	7 (41)
Number of lymph nodes harvested	8 (1–18)
Number of positive lymph nodes	1 (1–5)
LPLND specimen volume (mm^3^)	48,000 (7200–115,000)
Tumour size (mm)	40 (30–60)
Histology - Adenocarcinoma - Squamous cell carcinoma - Castleman’s disease (hyaline vascular type)	15 (88) 1 (6) 1 (6)
Tumour grade - Well to moderately differentiated - Poorly differentiated	16 (100) 0 (0)
Rectal tumour resection margin R0 R1/2	11 (100) 0 (0)

**Table 3:** Relationship between pathological characteristics and LPLN positivity status. Data presented as median (range). *: *p* < 0.05; ¥: *n* = 2; ƛ: *n* = 9.

	LPLN Positive (*n* = 7)	LPLN Negative (*n* = 10)
LN size, mm	14 (12–15) *	8 (4–15) *
Tumour height	5 (5–6) ^¥^	7 (4–10) ^ƛ^
Neoadjuvant treatment (yes/no)	0/2 ^¥^	5/4 ^ƛ^
Tumour size	30 (30–35) ^¥^	48 (30–75) ^ƛ^
EMVI/TD laterality positive (yes/no)	1/1 ^¥^	6/3 ^ƛ^

## Data Availability

The data presented in this study are available on request from the corresponding author. The data are not publicly available due to ethical reasons.
